# Unique encounter of a branchial cleft cyst compressing the great auricular nerve in an adult: a case report

**DOI:** 10.1093/jscr/rjae640

**Published:** 2024-10-10

**Authors:** Yazan AlHabil, Qasem N Dola, Liza Saadeddin, Haneen Sharabati

**Affiliations:** Faculty of Medicine and Health Sciences, Department of Medicine, An-Najah National University, Old Campus Street 7, Nablus 00970, Palestine; General Surgery, Jaffa Specialized Surgical Hospital, Yaffa Street, Ramallah 00970, Palestine; Faculty of Medicine and Health Sciences, Department of Medicine, An-Najah National University, Old Campus Street 7, Nablus 00970, Palestine; Faculty of Medicine and Health Sciences, Department of Medicine, Al-Quds University, University Street, Jerusalem 00970, Palestine

**Keywords:** branchial cleft cysts, great auricular nerve, surgical rarities, surgical intervention

## Abstract

Branchial cleft cysts, arising from developmental anomalies of the branchial apparatus, represent a rare entity in adult populations. This case report presents a unique instance of a 32-year-old female with a second branchial cleft cyst prominently involving the great auricular nerve, an undocumented finding in the existing literature. The patient initially presented with progressive left-sided neck swelling, accompanied by localized pain radiating to the auricle and headaches, without systemic symptoms. Diagnostic imaging confirmed a cystic mass adjacent to vital neck structures, necessitating surgical intervention. Histopathological analysis post-excision confirmed typical features of a benign branchial cyst. The postoperative course was uneventful, with complete resolution of symptoms upon follow-up. This case capitalizes the diagnostic challenges and therapeutic considerations in managing branchial cleft cysts, particularly highlighting the exceptional involvement of the great auricular nerve. Further research is warranted to elucidate the pathogenesis and optimal management strategies for such atypical presentations.

## Introduction

Branchial cleft cysts result from developmental anomalies in the branchial apparatus during embryogenesis. Various theories have been proposed since 1832, initially suggesting that these cysts arise from incomplete obliteration of branchial clefts, as suggested by Ascherson [1]. Subsequent hypotheses included the persistence of pre-cervical sinus, cystic changes in cervical lymph nodes, and remnants of thymo-pharyngeal duct [1]. Currently, the prevailing theory asserts that inadequate involution of the third and fourth branchial arches, which are covered by the second arch, leads to entrapment and subsequent transformation of their epithelial linings into branchial cysts [1, 2].

Branchial cleft cysts originate from the first to the fourth pharyngeal clefts, typically appearing as cystic structures, fistulas, or islands of cartilage, most commonly as lateral neck masses [3]. They are characterized as painless fluid-filled formations that typically manifest during childhood and adolescence; their initial presentation in adulthood is exceedingly rare [4].

Cysts originating from the second or third pharyngeal clefts commonly appear along the anterior border of the sternocleidomastoid muscle (SCM) [5]. Positioned anatomically close to the jugular vein, carotid artery, and hypoglossal nerve, these cysts are in intimate proximity to vital neck structures [5, 6]. Regardless, complications involving adjacent structures are sporadically reported in isolated cases [6–9].

The interconnection between the great auricular nerve (GAN) and branchial cysts in adult populations remains undocumented in the existing literature. Herein, we present this rare occurrence in a 32-year-old female patient.

## Case report

A 32-year-old female patient presented to the general surgery clinic with left-sided neck swelling of 4 months’ duration. Initially gradual and slow in progression, the swelling had rapidly progressed over 2 weeks before presentation, accompanied by jaw pain that radiates to the auricle, and a left-sided throbbing headache. She denied fever, chills, weight loss, night sweats, or previous similar swellings, and had no signs of dyspnea, dysphonia, or syncopal attacks.

**Figure 1:**
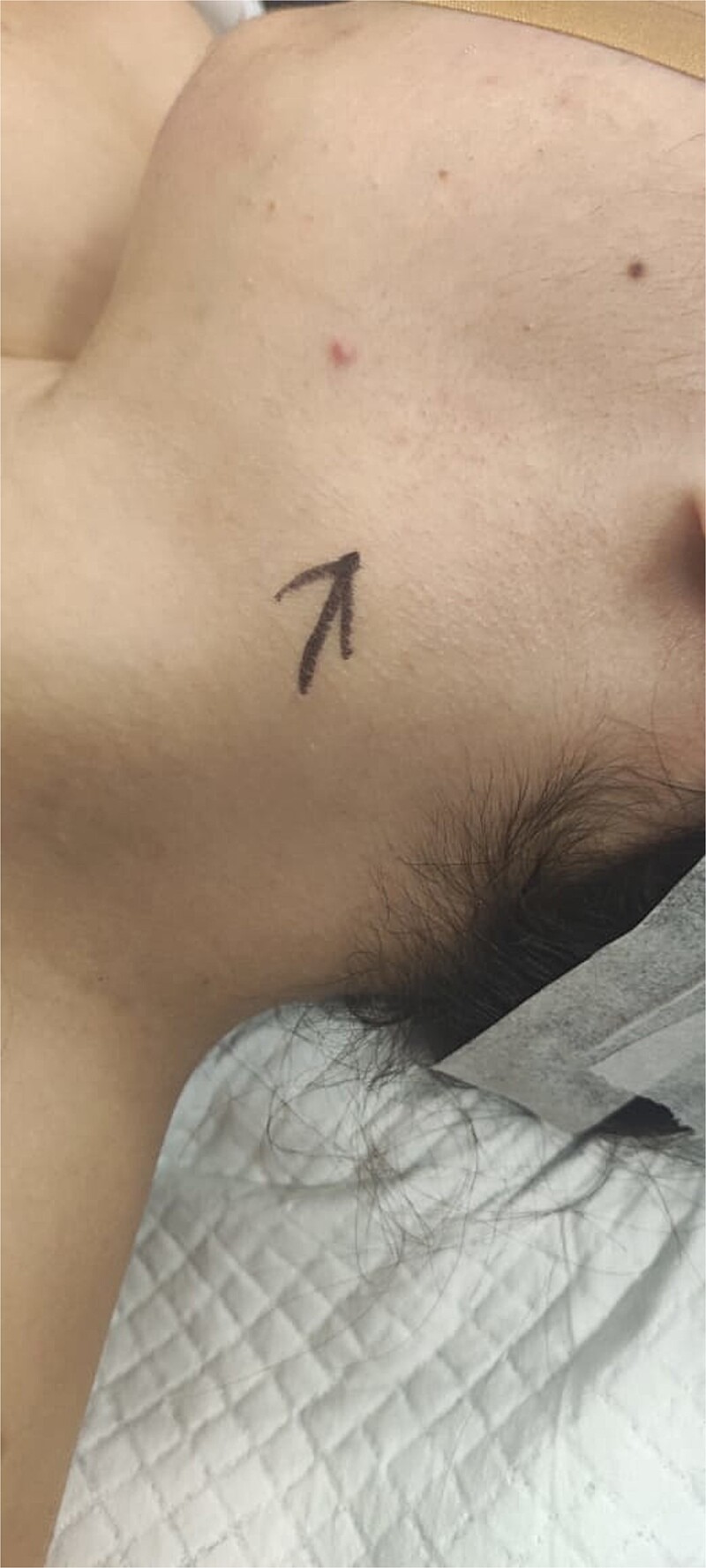
Gross photograph of the patient’s neck. Black drawn arrow points to the branchial cleft cyst.

On examination, the patient was conscious, alert, and oriented with stable vital signs. Head and neck examination revealed a single left-sided, oval neck swelling, measuring 4 × 2 cm on the upper part of the neck anterior to the SCM (Fig. 1). It had well-defined edges, normal overlying skin without erythema, and no visible or transmitted pulsations. The mass was not warm or tender to palpation, had a smooth surface, and was partially mobile, decreasing in size with SCM contraction. There were no enlarged cervical or axillary lymph nodes.

**Figure 2:**
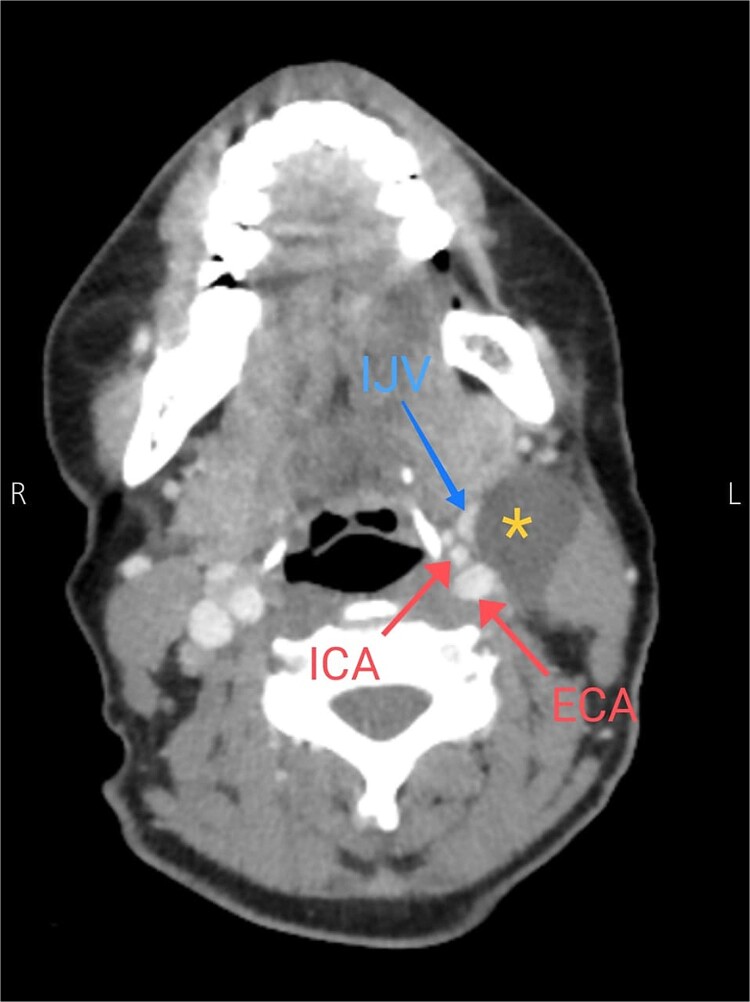
Axial CT neck scan showing the branchial cleft cyst compressing the IJV and adjacent to the external and internal jugular arteries. Asterisk: branchial cleft cyst, arrows: external and internal carotid arteries, blue arrow: IJV*.*

**Figure 3:**
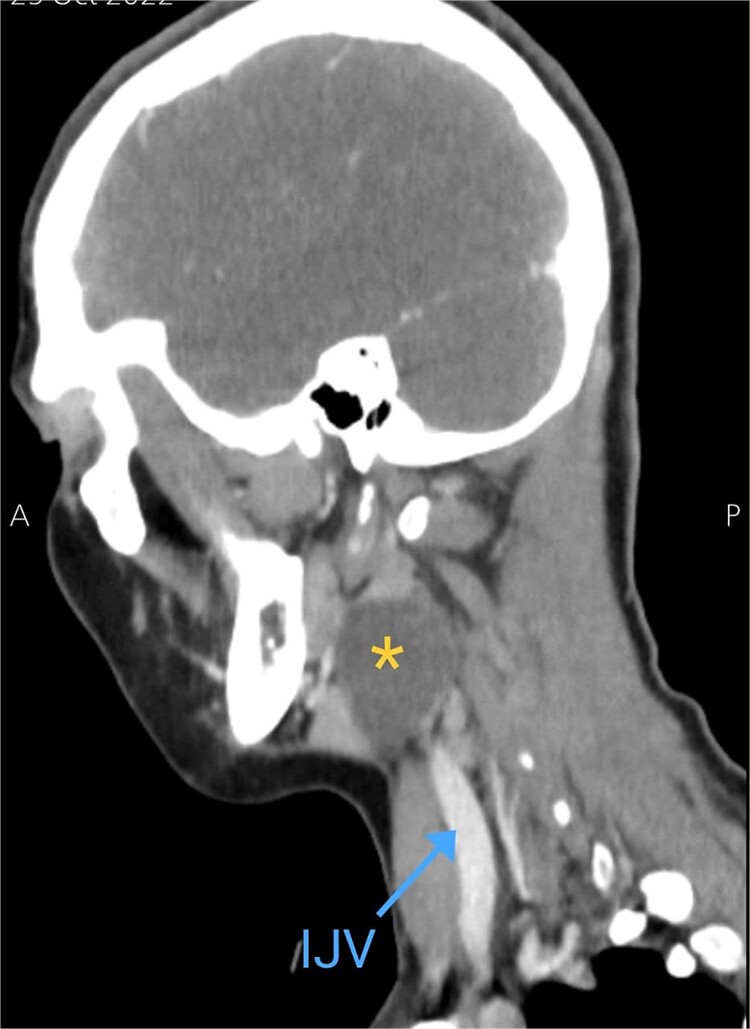
Sagittal CT neck scan showing the branchial cleft cyst. Asterisk: branchial cleft cyst, blue arrow: IJV.

**Figure 4:**
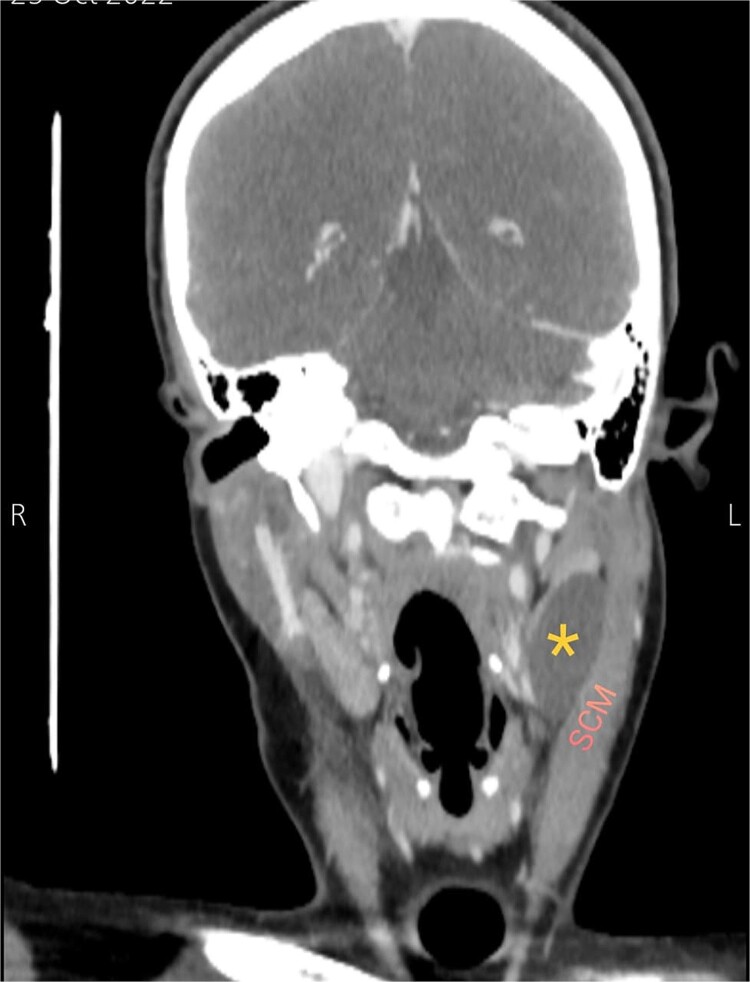
Coronal CT neck scan showing the branchial cleft cyst lying beneath the SCM. Asterisk: branchial cleft cyst.

Soft tissue ultrasound revealed a well-demarcated, oval-shaped, homogeneously hypoechoic with internal debris mass and posterior acoustic enhancement, measuring about 3.8 × 4.7 cm. Axial (Fig. 2), sagittal (Fig. 3), and coronal (Fig. 4) neck computed tomography (CT) scans with intravenous (IV) contrast confirmed the presence of a cystic mass measuring 4.2 × 3.1 cm with centered fluid density and thin walls, lying over the common carotid artery (CCA), internal carotid artery (ICA), and external carotid artery (ECA), partially compressing the internal jugular vein (IJV) and ECA coinciding with second branchial cleft cyst.

The patient was admitted to the hospital for surgical excision of the cyst. The patient, positioned supine under general anesthesia, underwent a left transverse submandibular neck incision overlying the cyst. Following meticulous scrubbing and draping, the incision was executed, and the wound was opened in layers to achieve the subplatysmal plane. The upper flap was dissected down to the mandibular level, while the lower flap was also elevated. The SCM was identified, with the cyst exerting posterior pressure on the SCM and compressing the GAN along its anterior trajectory over the muscle. Careful dissection of the cyst was performed, beginning posteriorly to separate it from the SCM. Multiple feeding vessels were identified and ligated. Complete detachment of the cyst from the SCM was achieved, extending to the prevertebral muscles. The dissection continued to delineate the cyst from the carotid sheath structures, including the IJV, CCA, and vagus nerve. The cyst was further dissected superiorly to its connecting duct, which was ligated, culminating in the total excision of the cyst. A Hemovac drain was inserted, and the wound was closed in layers.

**Figure 5:**
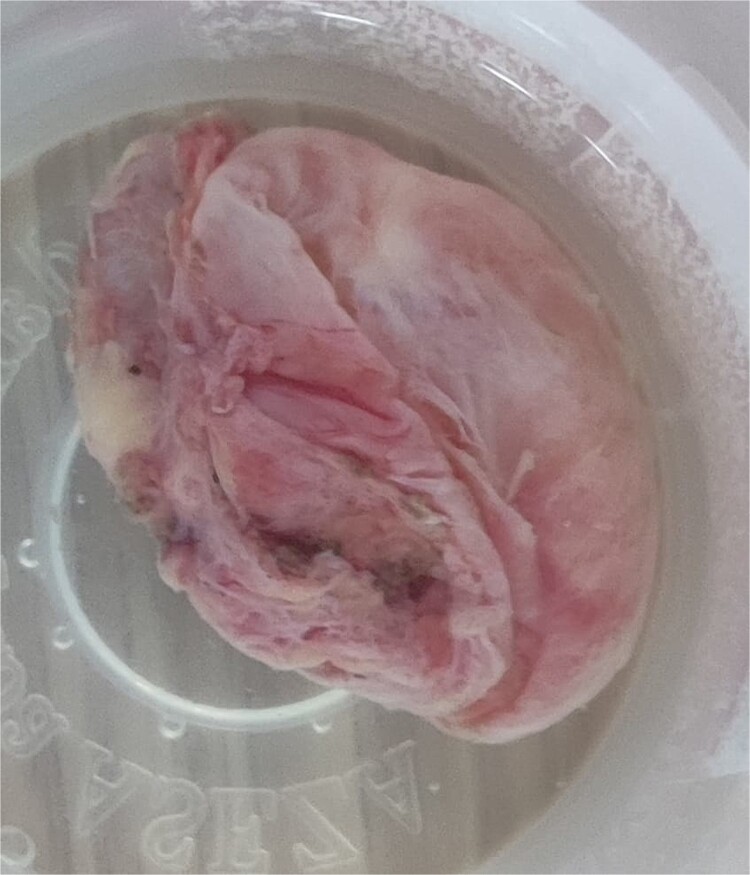
Gross photograph of the excised mass.

**Figure 6:**
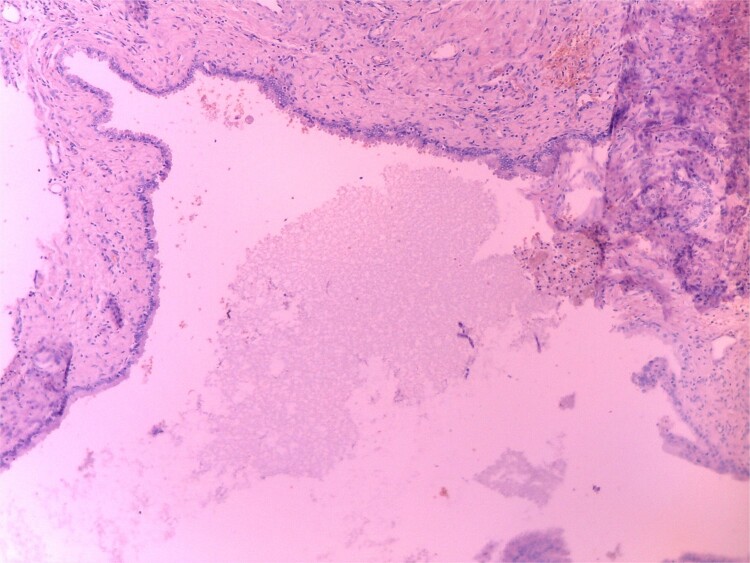
Microscopic histopathological picture showing a cystic structure lined by squamous and respiratory epithelium consistent with a branchial cleft cyst with no features of malignancy.

Histopathology revealed a cystic grayish mass measuring 3.5 × 2.5 × 1.2 cm on cut sectioning (Fig. 5), lined by squamous and respiratory epithelium with features consistent with a branchial cyst with no evidence of malignancy (Fig. 6).

The patient had a smooth postoperative course, with the drain removed the day following the surgery. She was discharged in good general condition. A follow-up 1 week postoperatively showed a well-healed wound and resolution of symptoms.

## Discussion

Branchial cleft cysts denote congenital anomalies in the embryogenesis of the four branchial clefts from the five ridges that contribute to the formation of six branchial arches [10]. The mesodermal ridges give rise to six branchial arches [10]. Externally, these arches form grooves or clefts, while internally they develop pouches [10]. Pouches are connected to a ventral or dorsal wing and lined with endoderm [10]. The derivatives of the branchial arches are lined with mesoderm, and ectoderm lines the branchial cleft structures [10]. Furthermore, branchial cleft cysts result from elements of the cervical sinus of His becoming entrapped without an external or internal opening, forming an epithelial-lined cyst [9]. They have also been proposed to develop from epithelial rests of tissue from Waldeyer’s ring. Each arch is associated with a cartilaginous bar, a derivative of the carotid artery, a cranial nerve, and a vein. In the first five arches, the nerve runs anterior to the associated artery; the sixth arch runs posterior to the artery [9, 11]. Various imaging methods such as ultrasound and CT imaging are used to diagnose branchial cleft cysts. Treatment involves complete surgical excision [10]. Differential diagnoses include hemangioma, ectopic thyroid or salivary gland tissue, and lymphadenopathy [10].

Diagnosing branchial cleft cysts is challenging due to their resemblance to other neck masses including hemangiomas, ectopic thyroid or salivary gland tissue, neurogenic tumors, and lymphadenopathy [10]. Ultrasound is often the first-line imaging modality, followed by CT or MRI for accurate mapping of the lesion and malignancy assessment [12]. Definitive diagnosis requires fine-needle aspiration biopsy or histopathological evaluation [12]. Treatment involves complete surgical excision, with infections addressed before surgery [10]. Surgical challenges include the risk of damaging vital structures such as the carotid artery, jugular vein, and cranial nerves [12]. In our case, the cyst’s compression of the GAN complicated the procedure, requiring careful dissection to avoid nerve damage and ensure complete removal.

Branchial cleft cysts are typically benign, painless, non-tender, and fluctuant masses in the lateral neck [6]. Involvement of adjacent neurovascular structures is exceedingly rare [6]. Long *et al.* proposed that such involvements occur following an upper respiratory tract infection that can transform a cyst into a tender and enlarged structure that causes nerve palsies and vessel thrombosis [6]. Rijuneeta *et al.* reported a case of IJV thrombosis secondary to a second branchial cyst in a 54-year-old male patient, following recurrent infections [7]. Gatot *et al.* and Long et al. reported the involvement of the hypoglossal nerve due to branchial cleft cysts [6, 8]. Additionally, Shin et al. reported recurrent nerves IX, X, and XII palsies in a 35-year-old within the context of a second branchial cleft cyst [9]. Tsur *et al.* reported transient facial nerve palsies following branchial cleft cyst excision in three pediatric patients [12].

Interestingly, we appear to be the first to document the involvement of the GAN secondary to a second branchial cleft cyst in a 32-year-old female patient without predisposing infectious processes or prior neurological symptoms. We hope that this case raises awareness among healthcare professionals and highlights the importance of further research to fully comprehend this uncommon occurrence.

## Data Availability

All patient-related data (history, findings, images, management, etc.) are all included in this manuscript.
